# Heart Failure With Reduced Ejection Fraction: A Retrospective Cross-Sectional Study of 40 Patients Referred to the Heart Failure Unit at San Juan de Alicante University Hospital

**DOI:** 10.7759/cureus.92714

**Published:** 2025-09-19

**Authors:** Carmen Bárbara Alfonso García, Idalberto Luis Fernandez Eng, Vicente Bertomeu, Eliany Leon Figueredo, Jorge Eugenio Sesin Hernandez, Misleydi Dominguez

**Affiliations:** 1 General Practice, Centro de Salud Benalua, Alicante, ESP; 2 Emergency Medicine, Hospital Universitario de la Ribera, Valencia, ESP; 3 Cardiology, Hospital Universitario San Juan de Alicante, Alicante, ESP; 4 General Medicine, University of Medical Sciences Cienfuegos, Cienfuegos, CUB; 5 Endocrinology, Diabetes and Metabolism, Albert Einstein College of Medicine, São Paulo, BRA; 6 General Medicine, Carlos J Finlay University, Camaguey, CUB

**Keywords:** guideline-directed medical therapy, heart failure, heart failure unit, left ventricular ejection fraction, retrospective study

## Abstract

Introduction/background

Heart failure (HF) is a prevalent clinical syndrome in Spain, often resulting in hospitalizations and significant morbidity. Guideline-directed medical therapy (GDMT) for HF with reduced ejection fraction (HFrEF) is strongly recommended to reduce mortality and improve cardiac function, yet it remains underutilized at hospital discharge. Structured HF Units (HFUs) may improve adherence to GDMT and optimize patient outcomes.

Methods

We conducted a retrospective, descriptive, cross-sectional study including 40 consecutive patients with HFrEF discharged from San Juan de Alicante University Hospital and referred to the HFU. At the HFU visit, patients underwent standardized assessments including medical history review, physical examination, New York Heart Association (NYHA) functional class evaluation, medication optimization according to GDMT, laboratory testing as needed, and repeat echocardiography when indicated. All consecutive eligible patients were included; no formal sample size calculation was performed. Quantitative variables were summarized as mean ± standard deviation and range, and qualitative variables as counts and percentages. Changes in left ventricular ejection fraction (LVEF) were analyzed using the Wilcoxon signed-rank test, while associations with age, sex, and GDMT completeness were evaluated using Spearman’s correlation and Mann-Whitney U tests (p < 0.05).

Results

The mean age of the cohort was 68.1 years (range 41-86), with a predominance of male patients (34 (85%)). At discharge, only eight (20%) received full GDMT, but this proportion more than doubled to 21 (52.5%) after follow-up in the HFU. This improvement was accompanied by a significant rise in mean LVEF from 31.5% to 43.0% (mean increase 11.6%, p < 0.001), with a greater gain observed in men. These findings clearly illustrate that the initially low prescription rate of GDMT at hospital discharge was significantly improved after HFU follow-up, and this optimization was associated with better cardiac function.

Conclusion

GDMT remains frequently underprescribed at hospital discharge, representing a missed opportunity for early optimization. Referral to a structured HFU significantly increases GDMT adherence and improves LVEF, underscoring the critical role of HFUs in multidisciplinary HF care.

## Introduction

Heart failure (HF) is a clinical syndrome characterized by symptoms and/or signs caused by a structural and/or functional cardiac abnormality, corroborated by elevated levels of natriuretic peptides and/or objective evidence of pulmonary or systemic congestion, as assessed via imaging or invasive hemodynamic measurements [1]. Left ventricular ejection fraction (LVEF) is the cornerstone for categorizing HF, guiding both therapeutic decisions and prognosis: HF with reduced ejection fraction (HFrEF): LVEF < 40%; HF with mildly reduced ejection fraction (HFmrEF): LVEF 41%-49%; and HF with preserved ejection fraction (HFpEF): LVEF ≥ 50%. In HFmrEF and HFpEF, confirmation of elevated filling pressures by natriuretic peptides or invasive hemodynamic techniques is required. Patients with previously reduced LVEF who improve to >40% are described as having improved LVEF [2]. Functional status is graded by the New York Heart Association (NYHA) from Class I (no limitation) to Class IV (symptoms at rest) [3]. HF can be acute, such as decompensated HF, acute pulmonary edema, right ventricular failure, or cardiogenic shock, or chronic, which may relapse into decompensation [4].

According to the Heart Failure Association (HFA) Atlas of the European Society of Cardiology (ESC), the median global HF prevalence is 17.2 per 1,000 individuals; in Spain, incidence is 2.76 per 1,000 person-years and prevalence 12 per 1,000 [5]. The HF-PATHWAYS study estimated in 2019 a prevalence of 1.89% and incidence of 2.78 per 1,000 person-years in Spain, with approximately 50% of cases being HFrEF and a higher prevalence in men due to ischemic heart disease [6,7]. Ischemic heart disease is the leading cause of acute HF admissions in high-income regions and Central/Eastern Europe [8,9].

HF typically begins as a consequence of reduced cardiac output and impaired pumping capacity of the heart. This decline activates several compensatory mechanisms, including the renin-angiotensin-aldosterone system (RAAS) and the sympathetic nervous system, that initially restore cardiovascular function. However, persistent activation over time contributes to ventricular damage and left ventricular remodeling, ultimately leading to cardiac decompensation [10].

Classical symptoms of HF include dyspnea, fatigue, and congestion-related manifestations such as orthopnea, paroxysmal nocturnal dyspnea, peripheral edema, and ascites. Physical examination may reveal tachypnea, Cheyne-Stokes respiration, and cardiac murmurs. Specific signs of volume overload include jugular venous distension, positive hepatojugular reflux, and bilateral pulmonary crackles [11]. Diagnostic evaluation begins with clinical suspicion, measurement of natriuretic peptides (N-terminal pro-B-type natriuretic peptide (NT-proBNP) or brain natriuretic peptide (BNP)), and confirmation by echocardiography, which also enables LVEF-based phenotyping [2].

Current guidelines advocate early initiation of four foundational HFrEF drug classes-angiotensin-converting enzyme inhibitors (ACEIs) or angiotensin receptor-neprilysin inhibitors (ARNIs), beta-blockers, mineralocorticoid receptor antagonists (MRAs), and sodium-glucose co-transporter 2 inhibitors (SGLT2i)-as first-line therapy in the absence of contraindications [12]. Indicated interventions such as valve procedures, atrial fibrillation ablation, or device implantation should not be delayed. Despite strong evidence that this four-pillar guideline-directed medical therapy (GDMT) strategy reduces HF hospitalizations and mortality, it remains underutilized; for example, in a US study (2015-2017), only 1% of patients received the full regimen, and 86% were not prescribed an ARNI [13].

In May 2020, the Cardiology Department at San Juan de Alicante University Hospital (HUSJ), Spain, established a dedicated HF Unit (HFU) for outpatient follow-up of patients discharged after decompensated chronic HF or newly diagnosed acute HF. Initially operating one day per week, the HFU expanded by October 2022 to six medical consultation days per month with continuous nursing support. Despite the availability of structured follow-up, the extent to which GDMT is optimized and whether LVEF improves after referral to the HFU remain unclear.

Despite strong evidence supporting GDMT in HFrEF, it is often underutilized at hospital discharge. Specialized HFUs provide structured follow-up that may optimize therapy and improve cardiac function. This study aimed to evaluate GDMT prescription at hospital discharge and to assess whether referral to a structured HFU improves adherence to therapy and LVEF in patients with HFrEF at HUSJ. We conducted a retrospective, descriptive, cross-sectional study including patients referred to the HFU to address these objectives.

## Materials and methods

Study design and setting

We conducted a retrospective, cross-sectional, descriptive study including 40 consecutive patients diagnosed with HFrEF who were discharged from HUSJ and later referred to the HFU between May 2022 and November 2022.

Rationale for the study design

The study is retrospective because it involved a review of existing medical records, cross-sectional because patient characteristics were evaluated at the time of referral without longitudinal follow-up beyond the study period, and descriptive because the primary objective was to report clinical features, management, and early outcomes rather than to evaluate a specific intervention.

Participants

Inclusion criteria were age ≥ 18 years, HFrEF defined as LVEF ≤ 40% measured by transthoracic echocardiography, and referral to the HFU. Patients who had died at the time of sample selection or those with preserved LVEF were excluded. Age and sex were recorded as standard baseline characteristics, since they influence clinical presentation, treatment strategies, and prognosis in HFrEF, allowing assessment of the cohort’s generalizability.

HFU procedures and data collection

At the HFU visit, patients underwent a structured evaluation. This included a review of medical history, physical examination, medication review and optimization according to current guidelines, laboratory tests, repeat echocardiography if clinically indicated, and patient education on adherence, self-monitoring, and follow-up planning.

A database was created in Microsoft Excel 2013 (Microsoft Corp., Redmond, WA, US) to record all patient information. Data collection was performed by the principal author without including any personal identifiers or clinical record numbers. Only the relevant sections of medical records were reviewed to extract study variables, which included demographics, LVEF, and discharge medication.

Sample size

All consecutive patients meeting the inclusion criteria were included. No formal sample size calculation was performed.

Statistical analysis

Statistical analyses were performed using IBM^®^ SPSS^®^ Statistics, version 26 (IBM Corp., Armonk, NY, US). Continuous variables were tested for normality using the Shapiro-Wilk test. Normally distributed variables are presented as mean ± standard deviation (SD), while non-normally distributed variables are expressed as median (interquartile range (IQR)). Categorical variables are reported as counts and percentages. Changes in LVEF were analyzed using the Wilcoxon signed-rank test, with effect size calculated as r = Z/√(n1 + n2). Associations between LVEF changes and age were evaluated with Spearman’s rank correlation coefficient (ρ), and relationships with sex and the complete discharge regimen from the HFU were assessed using the Mann-Whitney U test, with effect size calculated as r. A significance level of 5% was applied.

Ethics

The study was approved by the Research Ethics Committee of HUSJ. The requirement for informed consent was waived due to the retrospective nature of the study.

## Results

The mean age of the cohort was 68.1 years (range 41-86), reflecting the typical age distribution of patients with HFrEF and allowing assessment of cohort generalizability. Thirty-four patients (85%) were male, consistent with the known male predominance in HFrEF populations.

At hospital discharge, eight patients (20%) were receiving full GDMT. Among the remaining patients, five (12.8%) were on a single therapeutic pillar, six (15.4%) on two, 19 (48.7%) on three, and one (2.5%) with no medical treatment. Following referral to the HFU, 21 patients (52.5%) were on full GDMT, two (5%) on a single pillar, 10 (25%) on two pillars, and seven (17.5%) on three pillars. No patients remained without medical treatment after follow-up (see Table 1).

**Table 1:** Percentages of treatment regimens at hospital discharge and after referral to the HFU. One patient’s treatment regimen at hospital discharge was not available, explaining the total of 39 patients in this group. All 40 patients had data available after HFU referral. GDMT: guideline-directed medical therapy; HFU: Heart Failure Unit.

Treatment regimen	Hospital discharge N (%)	After HFU referral N (%)
Full GDMT	8 (20.0%)	21 (52.5%)
Three therapeutic pillars	19 (48.7%)	7 (17.5%)
Two therapeutic pillars	6 (15.4%)	10 (25.0%)
One therapeutic pillar	5 (12.8%)	2 (5.0%)
No medical treatment	1 (2.5%)	0 (0.0%)

**Figure 1:**
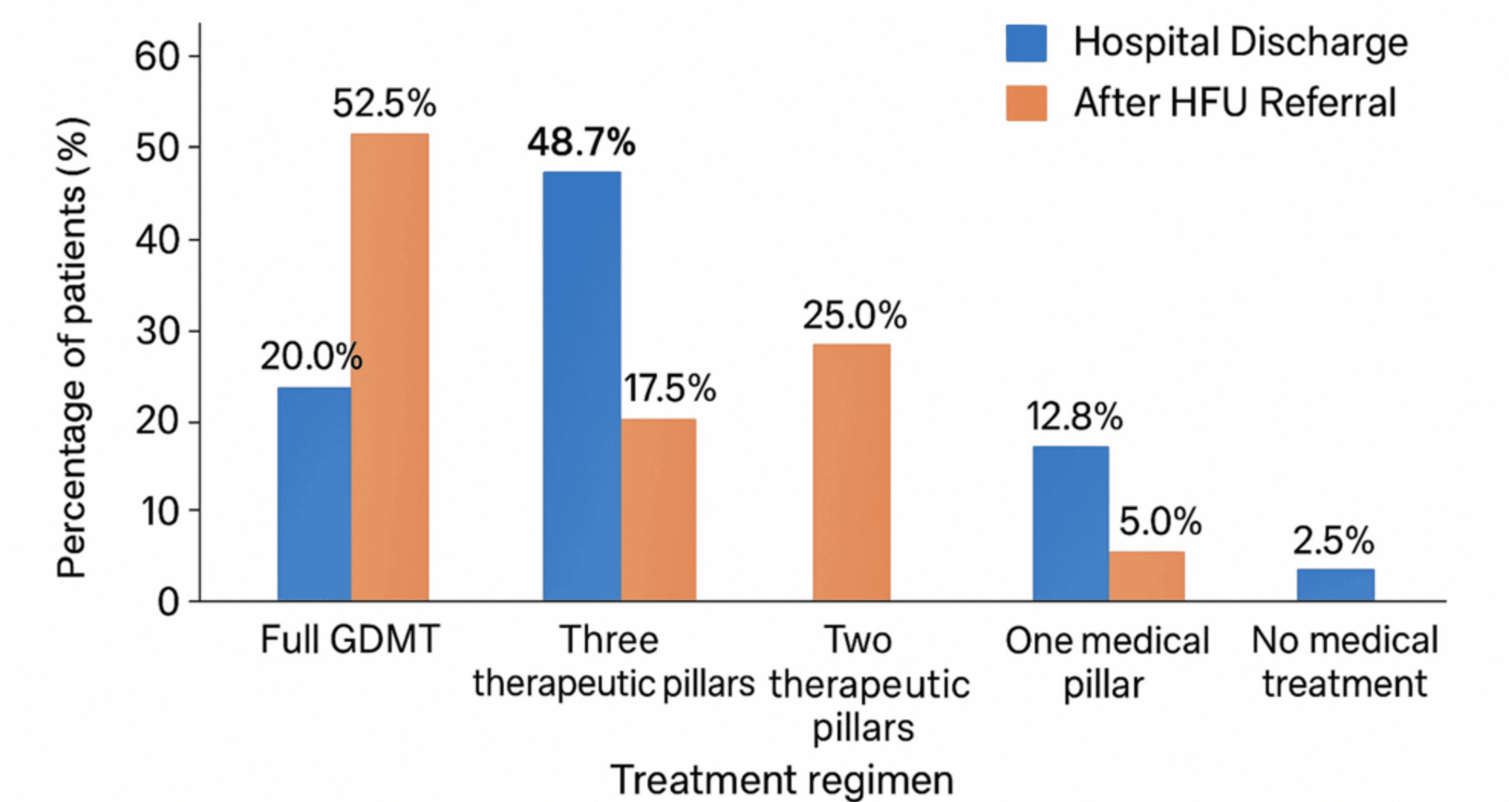
Percentage of patients treated according to medication group at hospital discharge and after follow-up in the HFU. One patient’s treatment regimen at hospital discharge was not available, explaining the total of 39 patients in this group. All 40 patients had data available after HFU referral. HFU: Heart Failure Unit; GDMT: guideline-directed medical therapy.

Beta-blockers were the most commonly used medication, prescribed to 33 patients (82.5%) at hospital discharge and increasing to 37 (92.5%) after HFU referral. Furosemide use decreased from 32 (80%) to 12 (30%). Angiotensin II receptor blockers (ARBs) decreased from 11 (27.5%) to five (12.5%), and ACEIs from four (10%) to two (5%). In contrast, SGLT2i increased from 19 (47.5%) to 33 (82.5%), eplerenone from 19 (47.5%) to 28 (70%), and ARNIs from 14 (35%) to 23 (57.5%) (see Figure 1).

The mean LVEF at hospital discharge was 31.5% (SD = 7.2), increasing to 43.0% (SD = 9.9) at the time of referral to the HFU. This represents an average improvement of 11.6 percentage points in LVEF (SD = 10.2), reflecting a significant enhancement in left ventricular function following treatment optimization. Table 2 summarizes the descriptive statistics for the variables analyzed. The mean age of the patients was 68.1 years, ranging from 41 to 86 years, with a median of 71 years, indicating a predominantly older population. The medians of LVEF at discharge and referral (31.5% and 44.0%, respectively) closely align with the means, suggesting a relatively symmetric distribution of the data.

**Table 2:** Descriptive statistics. SD: standard deviation; Min: minimum; Max: maximum; N: number of observations; %: percentage; HFU: Heart Failure Unit; LVEF: left ventricular ejection fraction.

Variable	Mean ± SD (Min–Max)	Median	N (%)
Continuous variables			
Age (years)	68.1 ± 12.3 (41–86)	71	–
LVEF at discharge (%)	31.5 ± 7.2 (20–47)	31.5	–
LVEF at follow-up (%)	43.0 ± 9.9 (20–60)	44	–
Change in LVEF (%)	11.6 ± 10.2 (-7–35)	11.5	–
Categorical variables			
Male sex	–	–	34 (85%)
Complete HFU regimen	–	–	21 (52.5%)

Concerning treatment, 21 (52.5%) patients received the complete recommended pharmacologic regimen upon admission to the HFU. These findings demonstrate a clinically meaningful improvement in cardiac function following specialized care in the HFU, a predominance of male patients in the cohort, and an increasing adherence to comprehensive pharmacologic therapy during follow-up.

**Figure 2:**
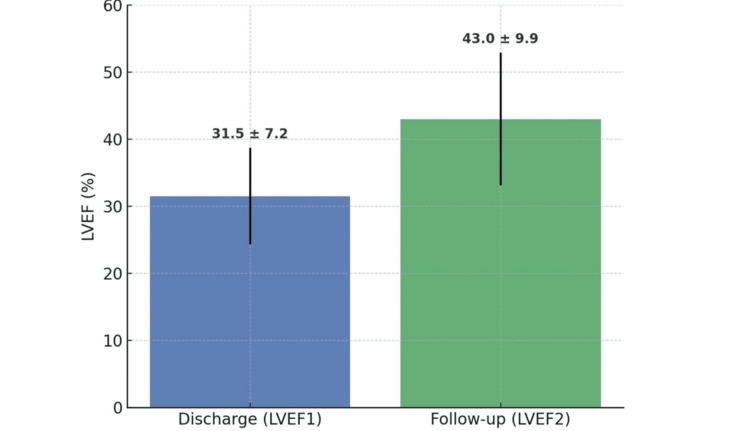
Left ventricular ejection fraction (LVEF) at hospital discharge and follow-up. LVEF1: measured at hospital discharge; LVEF2: measured after follow-up in the HFU (mean and standard deviation); HFU: Heart Failure Unit.

The Wilcoxon signed-rank test revealed a statistically significant increase in LVEF between the two time points, with a large effect size (Z = -4.967; p < 0.001; r = 0.79), as illustrated in Figure 2. This confirms a meaningful improvement in LVEF following patient referral to the HFU, along with their respective SDs, highlighting the substantial functional recovery observed.

**Figure 3:**
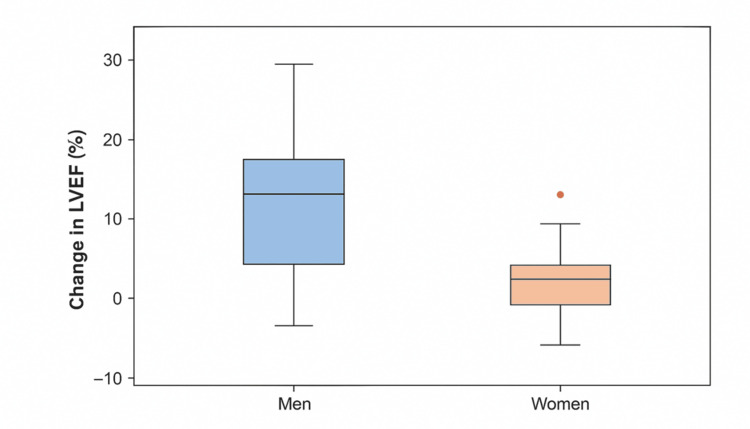
LVEF change in men and women. Distribution of LVEF change by sex. Boxplots show a higher median increase in men compared to women, with one female outlier. LVEF: left ventricular ejection fraction.

No significant correlation was observed between the change in LVEF and patient age (Rho = -0.106; p = 0.516). However, as shown in Table 3, male patients demonstrated a significantly greater mean increase in LVEF compared to female patients (13.0 ± 10.0 vs. 3.5 ± 7.6; p = 0.032; r = 0.34), corresponding to a moderate effect size. Figure 3 illustrates this difference, with men showing a broader distribution and higher median LVEF change, while women presented a narrower range and lower median values, including one identified outlier.

**Table 3:** Change in LVEF based on sex and complete discharge regimen in the Heart Failure Unit (UIC). M: mean; SD: standard deviation; rank: average rank; U: test statistic; p: significance level; r: effect size statistic.

Group	n	Mean (M)	SD	Rank	U	p	r
Gender							
Men	34	13.0	10.0	22.2	-	-	-
Women	6	3.5	7.6	11.1	45.500	0.032	0.34
High complete UIC schedule							
No	19	12.4	10.6	21.6	-	-	-
Yes	21	10.9	10.0	19.6	179.500	0.587	0.09

Conversely, no statistically significant difference was found in LVEF improvement between patients discharged from the HFU with the complete recommended medication regimen and those without it (12.4 ± 10.6 vs. 10.9 ± 10.0; p = 0.587; r = 0.09), suggesting that short-term LVEF recovery in this cohort was not directly influenced by medication completeness at discharge.

## Discussion

This descriptive study analyzed 40 patients referred to the HFU at HUSJ after hospitalization for acute decompensated or de novo HF, focusing on changes in LVEF, optimization of pharmacologic therapy, and patient distribution by demographic variables. The mean age of 68 years in our cohort was lower than that reported in larger epidemiological studies from Spain and Europe (72-78 years) [14-17], likely reflecting referral bias, as younger patients are more often admitted to HFU programs. The male predominance (85%) exceeded that reported in prior registries, such as Rachamin et al. [15] and Giner-Soriano et al. [17], where sex distribution was more balanced, but aligns with reports that men more frequently present with reduced LVEF [14,15].

At hospital discharge, only 20% of patients were prescribed full GDMT, consistent with real-world evidence showing that, even though hospitalization represents a key opportunity to initiate or continue therapy, many patients remain undertreated in the early post-discharge period [2,18]. After structured follow-up at the HFU, the proportion of patients on full GDMT increased to over 50%, demonstrating how dedicated follow-up can substantially improve adherence to guideline-directed therapy. Notably, the marked increase in ARNI, SGLT2i, and MRA use reflects progressive alignment with contemporary guideline recommendations (AHA/ACC/HFSA; ESC) [2,12].

In parallel, we observed a significant improvement in mean LVEF (from 31.5% to 43.0%), consistent with the beneficial effects of optimized therapy reported in recent clinical trials and real-world studies [12,17]. In our cohort, LVEF improvement was greater in men, whereas no significant correlation was found with age or baseline GDMT status. This contrasts with reports suggesting a more pronounced benefit in women treated with sacubitril/valsartan [19], while other studies indicate a lack of clear consensus regarding sex-related differences in treatment response [20]. These discrepancies underscore the complexity of sex-related variations in HF therapy and highlight the need for larger studies, though our small sample size limits definitive conclusions.

This study has several limitations, including its retrospective, observational design and relatively small cohort, which may introduce selection bias and limit statistical power. Additionally, we only included patients with reduced LVEF, excluding those with preserved or mildly reduced LVEF.

## Conclusions

HFrEF continues to pose a major clinical challenge, and optimal initiation of GDMT remains critical for improving prognosis. However, our study showed that GDMT is frequently underutilized at the time of hospital discharge, despite this being a key opportunity to optimize care. Subsequent adjustments during follow-up were associated with significant improvement in LVEF, particularly among male patients. These findings underscore the rationale for structured, protocol-driven management beginning at discharge and highlight the importance of ensuring early initiation of the four foundational pillars of HF therapy to maximize long-term outcomes.
